# Comparison of Changes in Incisors Position in Cases Treated with Damon Self-Ligating and Conventional Fixed Appliances

**DOI:** 10.2174/1874210601812010275

**Published:** 2018-03-30

**Authors:** Adriana Candida Albuquerque Nogueira, Karina Maria Salvatore Freitas, Darwin Vaz de Lima, Fabrício Pinelli Valarelli, Rodrigo Hermont Cançado

**Affiliations:** 1 Department of Orthodontics, Uninga University Center, Maringa-PR-Brazil; 2 Department of Orthodontics, Uninga University Center, Cuiaba-MT-Brazil

**Keywords:** Orthodontic brackets, Corrective orthodontics, Malocclusion, Cephalometry, Damon self-ligating, Conventional orthodontic brackets

## Abstract

**Objective:**

This study aimed to compare the changes in maxillary and mandibular incisors position in cases treated with Damon self-ligating and conventional fixed appliances.

**Methods:**

The sample comprised 51 patients with Class I malocclusion, mild to moderate crowding, treated without extractions, divided into 2 groups: Group 1 consisted of 20 patients treated with Damon self-ligating appliance, with a mean initial age of 15.00 years, treated for a mean period of 2.01 years; and Group 2 comprised 31 patients treated with conventional fixed appliances, with a mean initial age of 14.98 years, treated for a mean period of 1.81 years. The initial and final cephalograms of each patient were measured. The intergroup comparisons were performed with independent t or Mann-Whitney tests.

**Results:**

Both groups showed a mild protrusion and a buccal inclination of the maxillary and mandibular incisors, with no statistically significant difference between them.

**Conclusion:**

The changes in maxillary and mandibular incisors position were similar between the groups treated with Damon self-ligating and conventional fixed appliances.

## 1 INTRODUCTION

Self-ligating brackets have received great attention nowadays, orthodontic studies and practice, as an alternative treatment to conventional orthodontic brackets. Many orthodontic manufacturing companies have already developed their models of self-ligating brackets and with that, it is important to prove the viability of these brackets through scientific studies, to clarify the advantages of this treatment option [1].

One of the main propositions of self-ligating brackets is a treatment that offers less friction during tooth moving that allows orthodontic movement to be accomplished using lighter forces causing smaller damage to adjacent tissues, smaller root resorption [2] and faster mechanics, helping to reduce total treatment time [3]. Though the mechanics, reduction of the maxillary and mandibular incisors protrusion and a significant increase in the transversal dimensions of the dental arches can occur.

Research about changes in arch form and differences in incisor protrusion in self-ligating and conventional brackets has been performed, but there is still no consensus regarding the differences [4-6]. A metanalysis investigating the efficacy of self-ligating brackets observed that mandibular incisors were about 1.5º less proclined in cases treated with self-ligating than in conventional brackets [7]. However, regarding maxillary incisors protrusion, no data was found in the literature comparing self-ligating and conventional brackets.

In order to clarify this issue, the aim of the present study was to compare the changes in the position of maxillary and mandibular incisors in cases treated with Damon self-ligating and conventional fixed appliances.

## 2 MATERIALS AND METHODS

### 2.1 Materials

The research ethics committee of Humans Research of the UNINGA Inga University Center, Maringa-PR-Brazil (CAAE: 35487014.7.0000.5220) has approved this study.

The sample calculation was based on an Alpha significance level of 5% (0.05) and a Beta of 20% (0.2) in order to reach the test power of 80%, to detect a mean difference of 2.5^o^ with a standard deviation of 2.8 for mandibular incisor inclination [7]. Based on that, the sample calculation showed that at least 20 patients were necessary for each group.

The sample was retrospective, and the initial and final cephalograms and initial dental casts were used of each patient.

#### 2**.1.1. Criteria for Sample Selection**

Only patients presenting the following prerequisites were selected:

1. Good oral and systemic health.
2. No dental absences.
3. Light degree of anterior crowding.
4. Class I Angle malocclusion treated nonextraction.
5. No skeletal discrepancy.
6. Normal growth pattern (equilibrated).
7. Absence of periodontal disease.
8. Absence of prosthetic rehabilitation.
9. Cases treated only orthodontically, without extractions.
10. Full orthodontic treatment documentation, including lateral cephalogram at the beginning and end of treatment and dental casts from the initial stage.

Only patients who presented the following were considered for the sample.

#### 2.1.2 Sample Characteristics

The sample comprised 51 patients, divided into two distinct groups, according to the type of appliance used during treatment:

Group 1, formed by 20 patients (12 female and 8 male), treated orthodontically with Damon self-ligating appliance, with the mean initial age of 15.00 (s.d.=6.41) and mean final age of 17.01 years (s.d.=6.66). Mean treatment time was 2.01 years (s.d.=0.73). Mean Little index was 4.63 mm (s.d.=3.32) for the maxillary arch and 4.86 mm (s.d.=2.52) for the mandibular arch. The archwire sequence used was: 0.014” CuNiTi, 0.014”x0.025” CuNiTi, 0.018”x0.025” CuNiTi, 0.017”x 0.025” TMA and 0.019”x0.025” stainless steel. The diagram of the stainless steel archwire was made individually after alignment of the dental arches with the 0.014”x 0.025” CuNiTi wire, with reference to the bite registry in wax 7. This group was treated in a dental clinic at UNINGA Inga University Center, Cuiaba-MT-Brazil, by a single orthodontist.

Group 2, formed by 31 patients (17 female and 14 male), treated orthodontically with conventional Straight-Wire, Andrew’s prescription synthesis brackets, with the mean initial age of 14.98 (s.d.=3.54) and mean final age of 16.79 years (s.d.=3.45). Mean treatment time was 1.81 years (s.d.=0.60). Mean Little index was 5.47 mm (s.d.=2.45) for the maxillary arch and 4.40 mm (s.d.=1.91) for the mandibular arch. The archwire sequence used in orthodontic treatment was 0.014” NiTi, 0.016” NiTi, 0.016”, 0.018”, 0.020” and 0.019”x0.025” stainless steel. The diagram of stainless steel archwires was based on the initial arch form of each patient individually. This group was treated by students with a supervisor in an orthodontics course at UNINGA Inga University Center, Maringa-PR-Brazil.

Since patients present Class I malocclusion, in both groups, use of intermaxillary elastics was only for intercuspation for finishing the occlusion. No interproximal stripping was performed. After removal of the fixed appliances, patients used a Hawley plate as retention in the maxillary arch and a bonded 3x3 in the mandibular arch.

### 2.2 Methods

#### 2.2.1 Lateral Cephalograms

Two lateral cephalograms were used for each patient, one from the beginning of the fixed orthodontics appliance treatment (T1) and other from the end (T2).

The cephalograms were scanned using the Microtek ScanMaker i800 (9600 x 4800 dpi, da Microtek International, Inc., Carson, CA, USA) table scanner and attached to a Pentium microcomputer. The images were transferred to Dolphin Imaging Premium 10.5 programme (Dolphin Imaging & Management Solutions, Chatsworth, CA, USA) through which the images were digitalized and the measurements were processed.

Magnification factors used, depending on the apparatus used to take the cephalogram, were: 6% e 9.8%.

Only measurements of maxillary and mandibular incisors and overjet and overbite were included. Cephalometric variables included Steiner, Tweed and McNamara’s analysis measurements.

#### 2.2.2 Study Models

To analyse mandibular anterior crowding in the study models, Little's irregularity index was applied.

Little's irregularity index is a quantitative score used to evaluate mandibular anterior teeth irregularities. It involves measuring the real linear distance of anatomical contact points of each lower incisor to the anatomical contact point of the adjacent tooth, where the sum of these irregularities represents the distance to which the contact points should be moved to reach alignment.

The measurements were made using a Mitutoyo digital paquimeter, duly calibrated and using original active parts.

#### 2.2.3 Method Error

To determine the methodological error, 15 initial and 15 final cephalograms were randomly selected. The cephalograms were retraced, and two measurements for the same variables were obtained, at different times. Dahlberg’s formula [8] was applied and it allows the estimation of the order of magnitude of casual errors. To analyse systematic errors, the dependent t test was applied [9].

#### 2.2.4 Statistical Analysis

In order to evaluate the normality of the cephalometric variables, the test of Shapiro-Wilk was used.

Independent t test was used to check the compatibility of initial and final ages and treatment time. The non-parametric Mann-Whitney test was used to verify intergroup compatibility of the maxillary and mandibular crowding.

For intragroup comparison of the initial and final stages of treatment, dependent t or Wilcoxon tests were used. Intergroup comparison of initial and final stages and treatment changes were performed with independent t or Mann-Whitney tests.

All tests were made using Statistica software (Statistica for Windows 6.0, Statsoft, Tulsa, Okla, EUA). Significance level adopted was of 5% (*p*<0.05).

## 3 RESULT

No systematic errors were detected and random errors varied from 0.31 mm in the Overjet to 1.78º in the IMPA, and were considered acceptable.

Treatment with Damon self-ligating brackets caused proclination of the maxillary incisors and protrusion and proclination of the mandibular incisors, as well as a decrease in overjet and overbite (Table **1**).

Treatment with conventional fixed appliances showed proclination and protrusion of maxillary and mandibular incisors, as well as a decrease in overjet and overbite (Table **2**).

Groups were compatible regarding ages, treatment time and Little irregularity index (Table **3**).

The intergroup comparison of Damon and conventional groups showed no statistically significant difference in the incisors changes in the initial and final stages of treatment (Tables **4** to **6**).

## 4 DISCUSSION

For a reliable comparison of treatment changes, similar malocclusions are important, as well as age of the patients and initial crowding, that were compatible between the groups.

Treatment with Damon system showed buccal inclination of maxillary incisors, and proclination and protrusion of mandibular incisors. According to Vajaria *et al.* [10], it is possible to find dental proclination in both arches but in mandibular incisors, such proclination is more pronounced. Reduction in overbite can also be considered as a dental effect of expansion because it is affirmed that each millimeter increased in intermolar distance causes an overbite reduction of 0.283 mm [11].

In conventional group, maxillary and mandibular incisors proclination and protrusion were reported. Such behaviour was more noticeable in mandibular incisors. Overjet and overbite were corrected as well as upper lip retrusion, due to dental alignment and levelling.

### 4.1 Intergroup Comparison

#### 4.1.1 Maxillary Dentoalveolar Component

Maxillary anterior teeth showed no significant difference between the groups at initial and final stages and during treatment, indicating that both Damon self-ligating and conventional appliances caused similar maxillary incisor proclination and protrusion [12]. This does not corroborate the theory that the light forces caused by self-ligating brackets are not capable of promoting incisors proclination and protrusion. Said result implies that the use of superelastic wires is not able to overcome the strength of perioral muscles, especially the orbicular and mental muscle of the mouth, that should produce a “lip-bumper” effect on the incisors [2].

#### 4.1.2 Mandibular Dentoalveolar Components

Regarding the mandibular arch, Pandis *et al*. [13] and Fleming *et al.* [12], described identical incisors proclination and increase in intercanine distance using both conventional and self-ligating brackets during alignment and levelling stage [14].

Both showed proclination and protrusion in mandibular incisors, therefore there was no significant difference between the groups.

Scott [6] observed, in his randomized study comparing treatment efficiency between conventional and Damon system brackets after dental alignment, the inclination and protrusion of mandibular incisors on both groups, which prove that the result of the present paper is corroborating the results of previous studies.

The treatment of mandibular crowding with no tooth extraction implies in an increase of arch perimeter, arch length and protrusion and buccal inclination of incisors. The effects of this treatment modality are similar when comparing self-ligating and conventional brackets [12, 13].

#### 4.1.3 Dental Relationships

It has been shown that Damon self-ligating brackets are not clinically more effective than conventional brackets during orthodontic treatment [6].

Both groups showed correction in overbite and overjet after treatment, which is exactly an effect of dental crowding correction. However, both presented decrease in the two variables.

The decrease in overbite happens through molar extrusion, probably due to intermolar distance increase, characteristic present when dental crowding is treated nonextraction both in Damon and conventional groups [11].

### 4.2 Clinic Considerations

Previous studies have indicated that both types of appliances correct malocclusion by proclination of incisors, however self-ligating brackets showed a reduction of 1.5° of proclination when compared to conventional brackets [15].

The results showed that alterations in incisors positioning happen in both groups, although they are more pronounced in the mandibular arch. Even though Damon System affirms that the light forces of their intelligent wires, associated to low attrition on the brackets, are not capable of causing proclination and protrusion of incisors, it has been proved that this proclination happens and that there was no statistically significant difference when compared to the group subject to conventional treatment.

## CONCLUSION

The intergroup comparison of Damon and conventional groups showed that there was no significant difference between the groups in the proclination and protrusion of incisors.

## ETHICS APPROVAL AND CONSENT TO PARTICIPATE

The research was approved by the Ethics Committee in Humans Research of the UNINGA Inga University Center, Maringa-PR-Brazil (CAAE: 35487014.7.0000.5220).

## HUMAN AND ANIMAL RIGHTS

No animals were used in this research. All research procedures followed were in accordance with the ethical standards of the committee responsible for human experimentation (institutional and national), and with the Helsinki Declaration of 1975, as revised in 2008.

## CONSENT FOR PUBLICATION

All patients signed informed consent in Portuguese.

## Figures and Tables

**Table 1:** Comparison between initial and final stages of Damon group (dependent T test or Wilcoxon non-parametric test) (N=20).

**Variables**	**Initial Stage T1**		**Final Stage T2**		***P***
	**Mean (Median)**	**s.d. (i.r.)**	**Mean (Median)**	**s.d. (i.r.)**	
**Maxillary Dentoalveolar Component**					
1-NA (mm)	5.14	2.73	6.10	2.76	0.129 ^€^
1.NA (º)	24.37	7.88	29.10	6.98	0.014* ^€^
1-Aperp (mm)	6.83	2.12	6.95	2.61	0.823 ^€^
**Mandibular Dentoalveolar Component**					
1-NB (mm)	5.99	2.66	6.99	2.42	0.010* ^€^
1.NB (º)	29.60 (30.30)	6.14 (5.05)	33.40 (35.20)	6.63 (7.90)	0.008* ^¥^
1-AP (mm)	3.56	2.27	4.73	1.94	0.003* ^€^
IMPA (º)	96.83 (99.25)	6.83 (8.15)	100.97 (101.90)	7.29 (8.70)	0.004* ^¥^
**Dental Relationships**					
Overjet (mm)	3.67 (3.65)	1.69 (2.35)	3.10 (2.85)	0.76 (0.75)	0.163 ^¥^
Overbite (mm)	2.61	1.77	1.85	0.72	0.049* ^€^

Mean and standard deviation (s.d.) for dependent T test; median and interquartile range (i.r.) for Wilcoxon’s test. * Statistical significance for *p*<0.05 ^€^ Dependent T test ^¥^ Non parametric Wilcoxon’s test

**Table 2:** Comparison between initial and final stages of conventional group (Dependent T test or Wilcoxon non-parametric test) (N=20).

**Variables**	**Initial Stage T1**			**Final Stage T2**		***P***
	**Mean (Median)**		**s.d. (i.r.)**	**Mean (Median)**	**s.d. (i.r.)**	
**Maxillary Dentoalveolar Component**						
1-NA (mm)	5.34 (4.60)	3.50 (3.30)		6.25 (6.00)	2.50 (3.40)	0.037* ^¥^
1.NA (º)	26.81 (26.20)	6.73 (11.00)		30.64 (28.80)	5.14 (7.10)	0.009* ^¥^
1-Aperp (mm)	7.45 (7.00)	2.66 (2.50)		7.90 (7.80)	1.97 (2.60)	0.124 ^¥^
**Mandibular Dentoalveolar Component**						
1-NB (mm)	5.21 (4.70)	2.19 (3.10)		6.45 (5.90)	2.42 (2.80)	0.000* ^¥^
1.NB (º)	29.61 (28.80)	6.15(11.40)		32.68 (32.30)	5.73 (8.30)	0.002* ^¥^
1-AP (mm)	3.27 (3.30)	2.59 (4.30)		4.54 (4.10)	2.18 (2.60)	0.000* ^¥^
IMPA (º)	98.40	7.17		101.46	7.75	0.001* ^€^
**Dental Relationships**						
Overjet (mm)	3.76	1.28		2.70	0.76	0.000* ^€^
Overbite (mm)	3.08 (2.90)	1.48 (2.10)		1.56 (1.40)	0.59 (1.00)	0.000* ^¥^

Mean and standard deviation (s.d.) for dependent T test; median and interquartile range (i.r.), for Wilcoxon’s test. * Statistical significance for *p*<0.05 ^€^ Dependent T test ^¥^ Non parametric Wilcoxon’s test

**Table 3:** Intergroup compatibility of the initial and final ages and treatment time (independent t test) and maxillary and mandibular little irregularity index (non-parametric Mann Whitney test).

**Variables**	**Group 1** **Damon** **(N=20)**		**Group 2 Conventional** **(N=31)**		***P***
	**Mean**	**s.d.**	**Mean**	**s.d.**	
Initial age (y)	15.00	6.41	14.98	3.54	0.989
Final age (y)	17.01	6.66	16.79	3.45	0.876
Treatment time (y)	2.01	0.73	1.81	0.60	0.281
Mx Little (mm)	4.63	3.32	5.47	2.45	0.059
Md Little (mm)	4.86	2.52	4.40	1.91	0.801

**Table 4:** Results of intergroup comparison at initial stage of treatment (T1) (Independent T test or Mann Whitney non parametric test).

**Variables**	**Group 1** **Damon** **(N=20)**		**Group 2** **Conventional** **(N=31)**		***P***
	**Mean (Median)**	**s.d. (i.r.)**	**Mean (Median)**	**s.d. (i.r.)**	
**Maxillary Dentoalveolar Component**					
1-NA (mm)	5.14 (5.05)	2.73 (2.80)	5.34 (4.60)	3.50 (3.30)	0.938 ^¥^
1.NA (º)	24.37	7.88	26.81	6.73	0.242 ^€^
1-Aperp (mm)	6.83 (6.05)	2.12 (3.65)	7.45 (7.00)	2.66 (2.50)	0.412 ^¥^
**Mandibular Dentoalveolar Component**					
1-NB (mm)	5.99	2.66	5.21	2.19	0.263 ^€^
1.NB (º)	29.60 (30.30)	6.14 (5.05)	29.61 (28.80)	6.15 (11.40)	0.595 ^¥^
1-AP (mm)	3.56	2.27	3.27	2.59	0.686 ^€^
IMPA (º)	96.83 (99.25)	6.83 (8.15)	98.40 (100.40)	7.17 (11.00)	0.354 ^¥^
**Dental Relationships**					
Overjet (mm)	3.67	1.69	3.76	1.28	0.831 ^€^
Overbite (mm)	2.61	1.77	3.08	1.48	0.316 ^€^

Mean and standard deviation (d.p.) for Independent T test; median and interquartile range (i.r.), for Mann-Whitney non parametric test. ^€^ Independent T test ^¥^ Mann-Whitney non parametric test

**Table 5:** Results of intergroup comparison at final stage of treatment (T2) (independent t test).

**Variables**	**Group 1** **Damon** **(N=21)**		**Group 2** **Conventional** **(N=24)**		***P***
	**Mean (Median)**	**s.d. (i.r.)**	**Mean (Median)**	**s.d. (i.r.)**	
**Maxillary Dentoalveolar Component**					
1-NA (mm)	6.10	2.76	6.25	2.50	0.836 ^€^
1.NA (º)	29.10 (30.45)	6.98 (10.50)	30.64 (28.80)	5.14 (7.10)	0.742 ^¥^
1-Aperp (mm)	6.95	2.61	7.90	1.97	0.144 ^€^
**Mandibular Dentoalveolar Component**					
1-NB (mm)	6.99 (7.50)	2.42 (3.70)	6.45 (5.90)	2.42 (2.80)	0.263 ^¥^
1.NB (º)	33.40	6.63	32.68	5.73	0.680 ^€^
1-AP (mm)	4.73 (5.05)	1.94 (2.90)	4.54 (4.10)	2.18 (2.60)	0.423 ^¥^
IMPA (º)	100.97	7.29	101.46	7.75	0.822 ^€^
**Dental Relationships**					
Overjet (mm)	3.10 (2.85)	0.76 (0.75)	2.70 (2.70)	0.76 (0.90)	0.084 ^¥^
Overbite (mm)	1.86 (1.65)	0.72 (1.10)	1.56 (1.40)	0.59 (1.00)	0.091 ^¥^

Mean and standard deviation (s.d.) for Independent T test; median and interquartile range (i.r.), for Mann-Whitney non parametric test. ^€^ Independent t test ^¥^ Mann-Whitney non parametric test

**Table 6:** Results of intergroup comparison between the initial and final stages of treatment (T2-T1) (independent t test or Mann-Whitney non parametric test).

**Variables**	**Group 1** **Damon** **(N=21)**		**Group 2** **Conventional** **(N=24)**		***P***
	**Mean (Median)**	**s.d. (i.r.)**	**Mean (Median)**	**s.d. (i.r.)**	
**Maxillary Dentoalveolar Component**					
1-NA (mm)	0.95 (1.05)	2.69 (3.10)	0.90 (1.50)	3.04 (3.00)	0.671 ^¥^
1.NA (º)	4.73 (1.35)	7.88 (13.20)	3.83 (4.70)	7.49 (10.60)	0.938 ^¥^
1-Aperp (mm)	0.11	2.27	0.44	2.48	0.631 ^€^
**Mandibular Dentoalveolar Component**					
1-NB (mm)	1.00	1.57	1.23	1.48	0.591 ^€^
1.NB (º)	3.80	5.56	3.06	4.77	0.614 ^€^
1-AP (mm)	1.16	1.54	1.27	1.59	0.815 ^€^
IMPA (º)	4.14	5.50	3.05	4.92	0.465 ^€^
**Dental Relationships**					
Overjet (mm)	-0.57 (-0.25)	1.53 (2.15)	-1.06 (-0.60)	1.47 (1.90)	0.239 ^¥^
Overbite (mm)	-0.75	1.60	-1.51	1.33	0.069 ^€^

Mean and standard deviation (s.d.) for Independent T test; median and interquartile range (i.r.), for Mann-Whitney non parametric test. ^€^ Independent t test ^¥^ Mann-Whitney non parametric test
